# Brain‐Derived Neurotrophic Factor, Sarcopenia and Digital Gait Characteristics in Older Adults: Insights Into the Brain–Muscle Axis

**DOI:** 10.1002/jcsm.70325

**Published:** 2026-06-30

**Authors:** Chi Zhang, Ye Liu, Ji Shen, Yushan Zhang, Ying Liu, Kangzhen Zhang, Guoqing Fan, Jiling Liao, Dapeng Dai, Ping Zeng, Jing Li, Hong Shi, Yuhui Chen, Shiwei Liu, Jie Zhang

**Affiliations:** ^1^ Department of Basic Innovation Research, Beijing Hospital, National Center for Gerontology; National Clinical Research Center for Gerontology; the Key Laboratory of Geriatrics of NHC; Beijing Key Laboratory of Aging Mechanism and Intervention Research on Aging‐Related Diseases; Institute of Geriatric Medicine Chinese Academy of Medical Sciences Beijing China; ^2^ Department of Geriatrics, Beijing Hospital, National Center for Gerontology; National Clinical Research Center for Gerontology; the Key Laboratory of Geriatrics of NHC; Institute of Geriatric Medicine Chinese Academy of Medical Sciences Beijing China; ^3^ Chinese Center for Disease Control and Prevention (Chinese Academy of Preventive Medicine) Beijing China; ^4^ Department of Geriatrics Nanjing Central Hospital Jiangsu China; ^5^ Department of Neurology, Beijing Hospital, National Center for Gerontology; National Clinical Research Center for Gerontology; the Key Laboratory of Geriatrics of NHC; Institute of Geriatric Medicine Chinese Academy of Medical Sciences Beijing China

**Keywords:** brain‐derived neurotrophic factor, brain–muscle axis, gait characteristics, sarcopenia

## Abstract

**Background:**

Brain‐derived neurotrophic factor (BDNF) is a core biomarker involved in the brain–muscle axis. This study aimed to investigate the associations of plasma BDNF with sarcopenia and multidimensional gait characteristics in older adults.

**Methods:**

We enrolled 646 adults aged 60 years and older (mean age = 70.46 years; 56.97% females), among whom 65 (10.06%) were diagnosed with sarcopenia according to the Asian Working Group for Sarcopenia 2019. All participants underwent body composition analysis, comprehensive physical function assessments (handgrip strength, 6‐m walk and five‐time sit‐to‐stand test), and wearable sensor‐based gait evaluation (including 14 parameters). Plasma levels of BDNF, along with multiple inflammatory and oxidative stress markers, were quantitatively measured. Multivariable regressions were conducted to examine the association of BDNF with sarcopenia and gait parameters, with adjustments for demographic characteristics, lifestyle factors, activities of daily living, cognitive function, depression and comorbidities.

**Results:**

Plasma BDNF levels were significantly lower in older adults with sarcopenia compared to those without [1.82 (0.62, 4.95) μg/L versus 4.12 (0.81, 11.46) μg/L, *p* = 0.003]. BDNF was significantly correlated with appendicular skeletal muscle mass index (*r* = 0.11, *p* = 0.018), handgrip strength (*r* = 0.27, *p* < 0.001), gait speed (*r* = 0.32, *p* < 0.001) and sit‐to‐stand time (*r* = −0.34, *p* < 0.001). After full adjustment for covariates, the odds ratio (OR) for sarcopenia of ln‐BDNF was 0.71 (95% CI: 0.63–0.81, *p* = 0.012). Ln‐BDNF was negatively associated with swing time (*β* = −7.833, *p* = 0.011), step time (*β* = −12.769, *p* = 0.016), and stride time (*β* = −0.026, *p* = 0.012); and positively associated with thigh acceleration (*β* = 0.066, *p* = 0.001), thigh swing work (*β* = 0.039, *p* < 0.001), ground reaction force (*β* = 0.083, *p* < 0.001), foot landing control force (*β* = 0.224, *p* < 0.001), toe‐off angle (*β* = 2.061, *p* < 0.001), step frequency (*β* = 1.615, *p* < 0.001) and stride length (*β* = 0.013, *p* < 0.001). Interleukin‐6, interleukin‐1*β*, superoxide dismutase and glutathione reductase were concurrently correlated with BDNF levels, sarcopenia status and gait parameters. Mediation analysis demonstrated that interleukin‐1*β* mediated 14.65% of the relationship between BDNF and sarcopenia.

**Conclusion:**

Higher BDNF levels are associated with a lower prevalence of sarcopenia and superior kinetic and spatiotemporal gait performance in older adults. Future experimental studies are needed to explore the potential mechanism by which BDNF regulates sarcopenia via neuroinflammation.

## 1 Introduction

Sarcopenia is a pathological condition characterized by the loss of skeletal muscle mass, accompanied by diminished muscle strength or physical performance, which increases susceptibility to chronic diseases and elevates the risk of disability, falls and mortality [1, 2]. The pathogenesis of sarcopenia involves a complex physiological network, including dysregulated protein metabolism, chronic inflammatory responses, oxidative stress and impaired neural regulation [3, 4]. Recent studies have shown growing interest in identifying novel blood biomarkers for sarcopenia, which may enhance the understanding of its pathogenic mechanisms and inform health management strategies [3, 5]. Established neuromuscular biomarkers of skeletal muscle decline, including C‐terminal agrin fragment and neurofilament light chain, have proven diagnostic value for sarcopenia in prior research [6, 7]. However, biomarkers that simultaneously reflect central nervous regulation, sarcopenia risk and fine motor function decline remain limited.

Brain‐derived neurotrophic factor (BDNF) is a key mediator within the brain–muscle axis. It plays a crucial role in neuromuscular repair by enhancing neuromuscular transmission and regulating the development of the neuromuscular junction (NMJ) through its high‐affinity receptor, Tropomyosin receptor kinase B (TrkB) [8]. Conversely, BDNF deficiency has been shown to impair mitochondrial function, contributing to muscle atrophy and inflammation [9]. Although BDNF is considered a potential biomarker of skeletal muscle health, critical gaps remain in the evidence linking it to sarcopenia and in the elucidation of its underlying mechanisms. Lower BDNF levels have been associated with the development and progression of sarcopenia, manifested as insufficient protein synthesis, accelerated degradation, mitochondrial dysfunction, exacerbated oxidative stress and impaired NMJ function [10, 11]. Animal studies have demonstrated that BDNF levels in the gastrocnemius muscle of aged rats are significantly lower than those in adult rats, indicating an age‐related decline in the regenerative capacity of skeletal muscle after injury [12]. Furthermore, BDNF is critical in myogenesis, directly influencing muscle repair and regeneration, and participates in regulating metabolic pathways such as insulin‐mediated glucose uptake and *β*‐oxidation [11]. Most previous studies support the notion that BDNF protects against muscle wasting via its neuromuscular actions. In patients with malnutrition and chronic kidney disease, those with sarcopenia exhibit lower circulating BDNF levels, which are significantly correlated with handgrip strength and physical performance [13, 14]. A Korean study also found that BDNF was inversely associated with the risk of physical frailty after adjusting for age and multiple comorbidities [15]. However, recent evidence has reported conflicting results. For instance, a study by Jedd et al. found that community‐dwelling older adults with sarcopenia had higher plasma BDNF concentrations than healthy controls [16]. Another study from Brazil reported increased BDNF levels with greater sarcopenia severity in older women, yet this finding was limited by a small sample and the absence of confounding factor adjustment [17]. These discrepant findings suggest an unclear role for BDNF in skeletal muscle, leaving it unresolved whether it acts through direct regulation or a compensatory response. Consequently, the relationship appears to be highly contingent on an individual's specific health status.

Gait performance directly reflects the neural innervation and motor function of lower limb skeletal muscles and is closely linked to the progression of sarcopenia. Compared to simple gait speed assessment, multidimensional gait analysis using wearable sensors can provide more comprehensive biomechanical information, including spatiotemporal, rhythmic and kinetic parameters. Although declined gait speed is a key diagnostic criterion for sarcopenia, it fails to capture underlying issues such as insufficient lower limb muscle strength and impaired coordination. Refined gait analysis can precisely identify sarcopenia‐related alterations, including reduced cadence, weakened ground reaction force, altered toe‐off angle and prolonged lower limb support time. Notably, abnormal BDNF expression is implicated in the lower limb motor dysfunction and gait impairments observed in patients with various neurological disorders, including multiple sclerosis, Parkinson's disease and Alzheimer's disease [18, 19]. A body of evidence has confirmed that BDNF plays a pivotal role in counteracting muscle atrophy and motor dysfunction through dual anti‐inflammatory and antioxidant mechanisms. However, whether this protective function translates to a direct association with gait performance remains unexplored. By activating TrkB signalling, BDNF signalling suppresses the NF‐κB pathway, thereby downregulating proinflammatory cytokines such as TNF‐*α* and IL‐6 that promote proteolysis [10]. Concurrently, it enhances the nuclear factor erythroid two‐related factor 2 (Nrf2) pathway, upregulating antioxidant enzymes including superoxide dismutase (SOD) and glutathione peroxidase (GPx) to mitigate oxidative stress by neutralizing reactive oxygen species (ROS) [20]. This coordinated protection of neuromuscular integrity positions BDNF as a promising biomarker of sarcopenia‐related gait alterations. Investigating this association could provide deeper insights into the pathophysiological processes driving muscle wasting.

To address these critical gaps, this study aims to comprehensively investigate the associations of plasma BDNF levels with both sarcopenia and multidimensional gait characteristics in older adults. Additionally, we seek to explore the mechanistic pathways through which specific inflammatory and oxidative stress biomarkers may underlie BDNF's role in the brain–muscle axis.

## 2 Methods

### 2.1 Study Population

From February to August 2023, a cross‐sectional study was conducted among 937 older adults from five communities in Dongcheng District, Beijing. Participants underwent comprehensive assessments, including a structured questionnaire survey, digital gait measurement, health evaluation and biological sample collection. The inclusion criteria were as follows: (1) age ≥ 60 years; (2) clear consciousness and adequate communication ability; (3) the absence of severe psychiatric disorders; and (4) voluntary participation with willingness to complete the questionnaire and physical examination. We subsequently excluded individuals who were unable to complete gait measurements due to limited mobility (*n* = 45), those with missing blood samples (*n* = 212), participants who did not complete sarcopenia assessment (*n* = 7) and those with aberrant gait data due to systematic errors (*n* = 27). Consequently, a total of 646 valid participants were included in the final analysis. The study protocol was approved by the Ethics Committee of Beijing Hospital (Approval No. 2022BJYYEC‐263‐02), and written informed consent was obtained from all participants.

### 2.2 Diagnosis of Sarcopenia

Appendicular skeletal muscle mass (ASM) was measured using bioelectrical impedance analysis (Inbody 770, South Korea). Skeletal muscle mass index (SMI) was calculated as ASM/height^2^ (kg/m^2^). Low muscle mass was defined as SMI < 7.0 kg/m^2^ for men and < 5.7 kg/m^2^ for women. Handgrip strength was measured using a handheld dynamometer. Low muscle strength was defined as grip strength < 28 kg for men and < 18 kg for women. Physical performance was evaluated using a comprehensive battery of tests, including usual gait speed measured over a 6‐m walking course, five times sit‐to‐stand test time, and the Short Physical Performance Battery (SPPB). Low physical performance was defined as either gait speed < 1.0 m/s, five times sit‐to‐stand time ≥ 12 s or SPPB score ≤ 9 points. According to the 2019 Asian Working Group for Sarcopenia (AWGS) consensus, sarcopenia was diagnosed by the presence of low muscle mass combined with low muscle strength and/or low physical performance [21].

### 2.3 Gait Measurement

**FIGURE 1:**
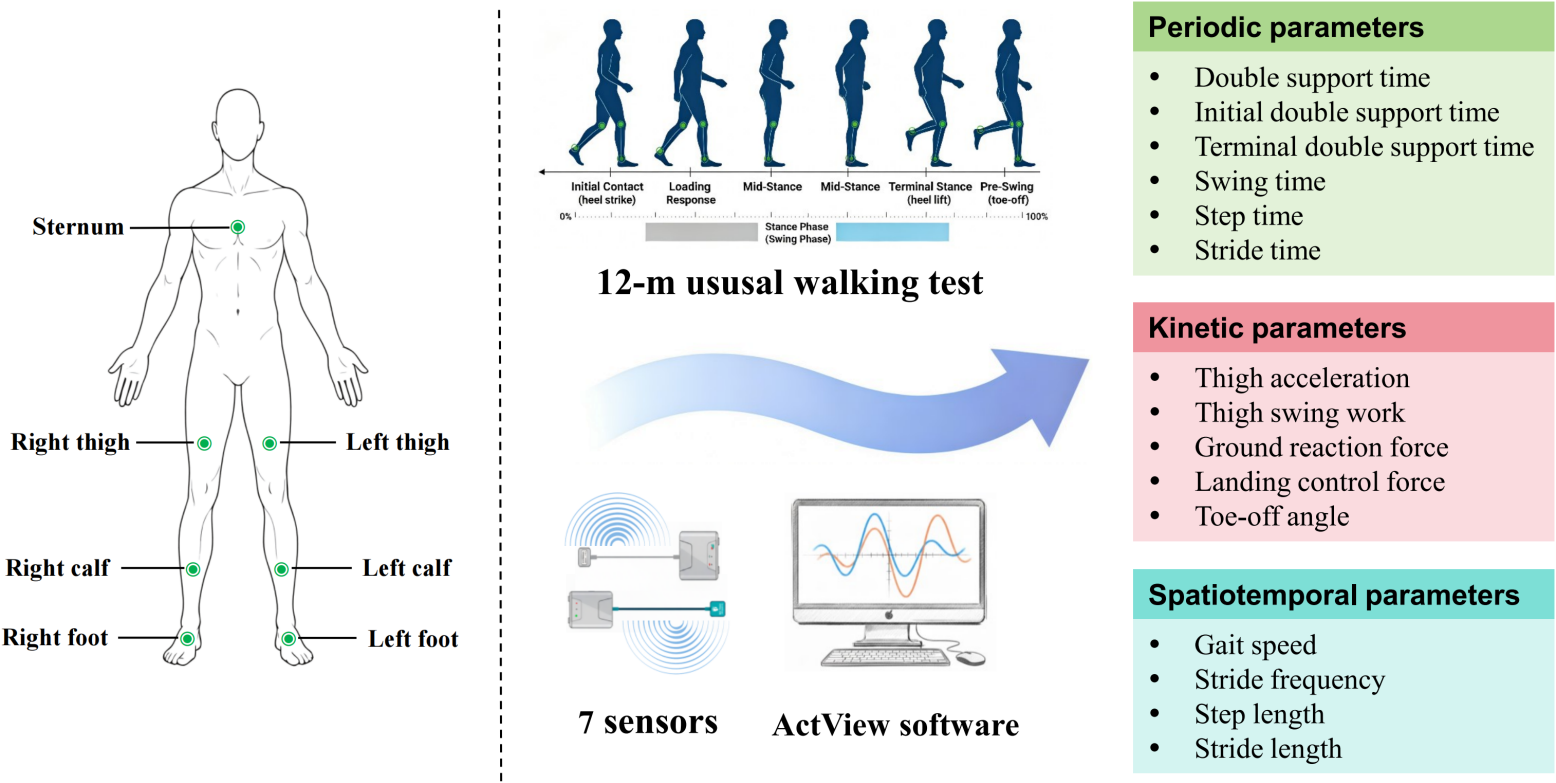
Procedure for measuring gait characteristics using wearable sensors.

A multisensor wearable system (IDEEA, MiniSun LLC, USA) was used for gait data acquisition. The system's main unit and seven sensors were deployed according to manufacturer guidelines, with their reliability, accuracy and repeatability having been well documented previously [22, 23]. Participants were instructed to walk 12 m at their habitual pace on level ground after being equipped with the sensors. The recorded data were transferred to a computer and processed using ActView software, from which 14 gait parameters were acquired, reflecting bilateral lower limb performance. The analysed metrics were categorized into periodic (double‐limb support time, initial limb support time, terminal limb support time, swing time, step time and stride time), kinetic (thigh acceleration, thigh swing work, ground reaction force, foot landing control force and toe‐off angle) and spatiotemporal parameters (stride frequency, step length and stride length). Figure 1 illustrates the procedure for collecting gait parameters.

### 2.4 Blood Sample

Blood samples were obtained from all participants between 7:00 and 9:00 AM before breakfast, following an 8–12 h overnight fast. All participants were asked to refrain from drinking alcohol, smoking and engaging in vigorous physical activity within 24 h before blood sampling. For each participant, 5 mL of venous blood was drawn into EDTA‐K2 anticoagulant tubes, followed by centrifugation at 3000 rpm for 10 min at 4°C to isolate platelet‐poor plasma, with the cellular fraction (including platelets, leukocytes and erythrocytes) completely discarded. The upper PPP layer was immediately aliquoted, transported under constant 2°C–8°C cold chain conditions to the Beijing Hospital biobank within 2 h of collection and stored at −80°C until batch analysis. All samples underwent no more than one freeze–thaw cycle before biomarker quantification to avoid protein degradation. The concentration of plasma BDNF was quantified using a flow‐based multiplex immunoassay (RayBiotech, USA; FAH‐CUST). The interassay and intra‐assay coefficients of variation (CV) were 2.7% and 4.2%, respectively. The accuracy of multiplex assays for quantifying plasma proteins has been well established in previous studies [24, 25]. Plasma BDNF levels exhibited a skewed distribution and were therefore natural‐log (ln)‐transformed for normality. Participants were categorized into tertiles of BDNF levels: low BDNF (< 1.46 μg/L), medium BDNF (≥ 1.46 to < 7.79 μg/L) and high BDNF (≥ 7.79 μg/L). To explore potential underlying mechanisms, a panel of circulating inflammatory and oxidative stress biomarkers was also assessed. These indicators included high‐sensitivity C‐reactive protein (hs‐CRP), interleukin‐6 (IL‐6), interleukin‐1 beta (IL‐1*β*), malondialdehyde (MDA), SOD, glutathione reductase (GR) and glutathione peroxidase (GSH‐Px). All markers were measured using commercially available enzyme‐linked immunosorbent assay (ELISA) kits (RayBiotech, USA). The inter‐assay and intra‐assay CVs for these analytes ranged from 1.9% to 7.1% and 2.6% to 8.6%, respectively.

### 2.5 Covariates

A series of covariates were considered in multiple analyses based on previous studies [16, 26, 27, 28]. Demographic characteristics (age, gender, ethnicity and marital status) and data on current smoking, current alcohol consumption and exercise habits were collected during face‐to‐face interviews by trained investigators. Habitual exercise was assessed per the physical activity component of the Fried frailty scale and dichotomized by sex‐specific thresholds, where men with weekly energy expenditure < 383 kcal (~2.5 h of walking) and women < 270 kcal (~2 h of walking) were classified as nonhabitual exercisers and those meeting or exceeding the thresholds were defined as habitual exercisers. Height and weight were measured to calculate body mass index (BMI). The Barthel Index was used to assess activities of daily living (ADL), with a total score < 95 indicating impaired ADL. Cognitive function was measured using the Mini‐Mental State Examination (MMSE). Mental health status was assessed using the Patient Health Questionnaire (PHQ‐9), with a total score > 4 indicating depressive symptoms. The Charlson Comorbidity index (CCI) was used to assess the presence of common chronic diseases.

### 2.6 Statistical Analyses

Continuous variables were presented as mean ± standard deviation (M ± SD) for normally distributed data or median with interquartile range [M (P_25_, P_75_)] for nonnormally distributed data. Categorical variables were described as frequency (percentage) [*n* (%)]. Differences in characteristics between the sarcopenia and nonsarcopenia groups were compared using Student's *t* test, Wilcoxon rank‐sum test or chi‐square test, as appropriate. All correlation analyses were performed using the Spearman correlation coefficient. Multivariable logistic regression was employed to examine the association between BDNF levels and sarcopenia, with results expressed as odds ratios (OR) and 95% confidence intervals (CI). Receiver operating characteristic (ROC) curve analysis was performed to assess the discriminatory value of circulating BDNF for sarcopenia, with the area under the curve (AUC) and 95% CI calculated, and the optimal cut‐off value determined using Youden's index. Multivariable linear regression was used to analyse the relationship between natural log‐transformed BDNF (ln‐BDNF) and gait parameters. To explore relevant biomarkers, we first performed pairwise Spearman correlation analyses to screen inflammatory and oxidative stress markers concurrently associated with BDNF levels, sarcopenia status and gait parameters. We then used partial least squares structural equation modelling (PLS‐SEM) to test the mediating role of the screened markers on the BDNF‐sarcopenia association. The significance of indirect effects was verified via bias‐corrected bootstrap with 1000 resamples to generate robust 95% CIs and a mediation effect was deemed significant when the 95% CI of the proportion mediated (PM) did not span 0. All analyses were conducted in R version 4.2.0. A two‐sided *p* < 0.05 was considered statistically significant.

## 3 Result

### 3.1 Sample Characteristics

The mean age of the 646 participants was 70.46 ± 6.13 years, and 56.97% were female. The median plasma BDNF concentration was 3.66 (0.81, 10.39) μg/L. According to the 2019 AWGS diagnostic criteria, 65 individuals (10.06%) were identified as having sarcopenia. Participants with sarcopenia were older, had greater impairments in ADL, lower cognitive function and a higher prevalence of depression and multimorbidity (all *p* < 0.05). Significant differences were also observed in kinetic and spatiotemporal gait parameters between the sarcopenia and non‐sarcopenia groups. Detailed demographic and clinical characteristics of the study participants are presented in Table 1.

**TABLE 1:** Demographic characteristics, sarcopenia status, gait parameters and biomarkers in 646 older adults.

Sample characteristics	Total sample *n* = 646	Nonsarcopenia *n* = 581	Sarcopenia *n* = 65	*p*
**Demographic features**				
Age (m ± SD, year)	70.46 ± 6.13	68.97 ± 5,79	74.22 ± 6.27	< 0.001
Female [*n* (%)]	368 (56.97)	327 (56.28)	41 (63.08)	0.297
Han ethnicity [*n* (%)]	604 (93.50)	544 (93.63)	60 (92.31)	0.634
Married and living together [*n* (%)]	536 (82.97)	487 (83.82)	49 (75.38)	0.083
Current smoking [*n* (%)]	78 (12.07)	73 (12.56)	5 (7.69)	0.239
Current drinking [*n* (%)]	77 (11.92)	70 (12.05)	7 (10.77)	0.789
Habitual exercise [*n* (%)]	560 (86.69)	507 (87.26)	53 (81.54)	0.185
BMI (m ± SD, kg/m^2^)	24.74 ± 3.51	25.03 ± 3.43	21.92 ± 3.01	< 0.001
ADL impairment [*n* (%)]	41(6.36)	33 (5.68)	8 (12.31)	0.036
MMSE (m ± SD)	27.64 ± 2.29	27.75 ± 2.15	26.58 ± 3.19	0.007
Depressive symptoms [*n* (%)]	84 (13.00)	70 (12.05)	14 (21.54)	0.032
Charlson index [M (P_25_, P_75_)]	2 (1,3)	2 (1,3)	2 (1,4)	0.037
**Sarcopenia conditions**				
ASMI (m ± SD, kg/m2)	6.91 ± 1.07	7.04 ± 1.02	5.74 ± 0.72	< 0.001
Handgrip strength (m ± SD, kg)	28.42 ± 9.28	29.04 ± 9.23	22.46 ± 7.62	< 0.001
6‐m gait speed (m ± SD, m/s)	1.05 ± 0.23	1.06 ± 0.23	0.97 ± 0.24	0.012
Five times chair stand test (m ± SD, s)	10.82 ± 3.46	10.65 ± 3.45	12.45 ± 3.06	< 0.001
SPPB (m ± SD)	9.41 ± 1.56	9.52 ± 1.51	8.25 ± 1.62	< 0.001
**Periodic parameters**				
Double‐limb support time (m ± SD, ms)	412.59 ± 63.08	412.29 ± 64.44	415.58 ± 48.53	0.631
Initial limb support time (m ± SD, ms)	132.18 ± 16.43	132.61 ± 16.46	128.03 ± 15.68	0.036
Terminal limb support time (m ± SD, ms)	152.67 ± 34.16	152.89 ± 34.38	150.56 ± 32.11	0.597
Swing time (m ± SD, ms)	425.96 ± 61.83	426.76 ± 63.23	418.42 ± 45.97	0.202
Step time (m ± SD, ms)	591.71 ± 108.75	593.59 ± 110.52	573.67 ± 88.79	0.111
Stride time (m ± SD, s)	1.22 ± 0.21	1.22 ± 0.21	1.18 ± 0.17	0.139
**Kinetic parameters**				
Thigh acceleration (m ± SD, G)	1.16 ± 0.41	1.17 ± 0.41	1.12 ± 0.42	0.394
Thigh swing work (m ± SD, G)	0.61 ± 0.21	0.63 ± 0.21	0.55 ± 0.17	0.006
Ground reaction force (m ± SD, G)	1.23 ± 0.39	1.25 ± 0.39	1.09 ± 0.32	0.001
Foot landing control force (m ± SD, G)	2.91 ± 0.96	2.94 ± 0.97	2.65 ± 0.83	0.016
Toe‐off angle (m ± SD,°)	25.53 ± 11.88	26.02 ± 11.96	20.93 ± 10.11	0.001
**Spatiotemporal parameters**				
Stride frequency (m ± SD, steps/min)	110.52 ± 14.09	110.27 ± 14.08	112.92 ± 13.98	0.167
Step length (m ± SD, m)	0.56 ± 0.07	0.57 ± 0.07	0.52 ± 0.06	< 0.001
Stride length (m ± SD, m)	1.03 ± 0.15	1.04 ± 0.15	0.97 ± 0.13	< 0.001
**Blood biomarkers**				
BDNF [M (P_25_, P_75_), μg/L]	3.66(0.81,10.39)	4.12(0.81,11.46)	1.82(0.62,4.95)	0.003
Hs‐CRP [M (P_25_, P_75_), mg/L]	1.47(0.67,3.29)	1.51(0.68,3.30)	1.38(0.78,3.51)	0.855
IL‐6 [M (P_25_, P_75_), pg./mL]	8.03(3.11,14.93)	12.03(1.56, 47.65)	23.09(9.65,64.28)	0.004
IL‐1*β* [M (P_25_, P_75_), pg./mL]	13.35(6.89,25.05)	12.66(3.27,20.48)	23.88(6.71,31.02)	0.023
MDA [M (P_25_, P_75_), nmol/mL]	3.51(1.04,7.22)	3.51(0.91,7.21)	3.49(1.22,6.49)	0.682
SOD (m ± SD, U/mL)	14.36 ± 5.09	14.27 ± 5.02	14.99 ± 5.35	0.058
GSH‐Px [M (P_25_, P_75_), U/mL]	195.45(122.18265.64)	197.64(122.02269.92)	195.02(138.75258.82)	0.785
GR [M (P_25_, P_75_), U/L]	10.87(3.49,30.87)	10.49(3.49,32.03)	9.80(2.91,21.55)	0.367

Abbreviations: ADL, activities of daily living; ASMI, appendicular skeletal muscle index; BDNF, brain‐derived neurotrophic factor; BMI, body mass index; GR, glutathione reductase; GSH‐Px, glutathione peroxidase; Hs‐CRP, high‐sensitivity C‐reactive protein; IL‐1*β*, interleukin‐1 beta; IL‐6, interleukin‐6; MDA, malondialdehyde; MMSE, mini‐mental state examination; SOD, superoxide dismutase; SPPB, short physical performance battery.

### 3.2 Association Between BDNF and Sarcopenia

**FIGURE 2:**
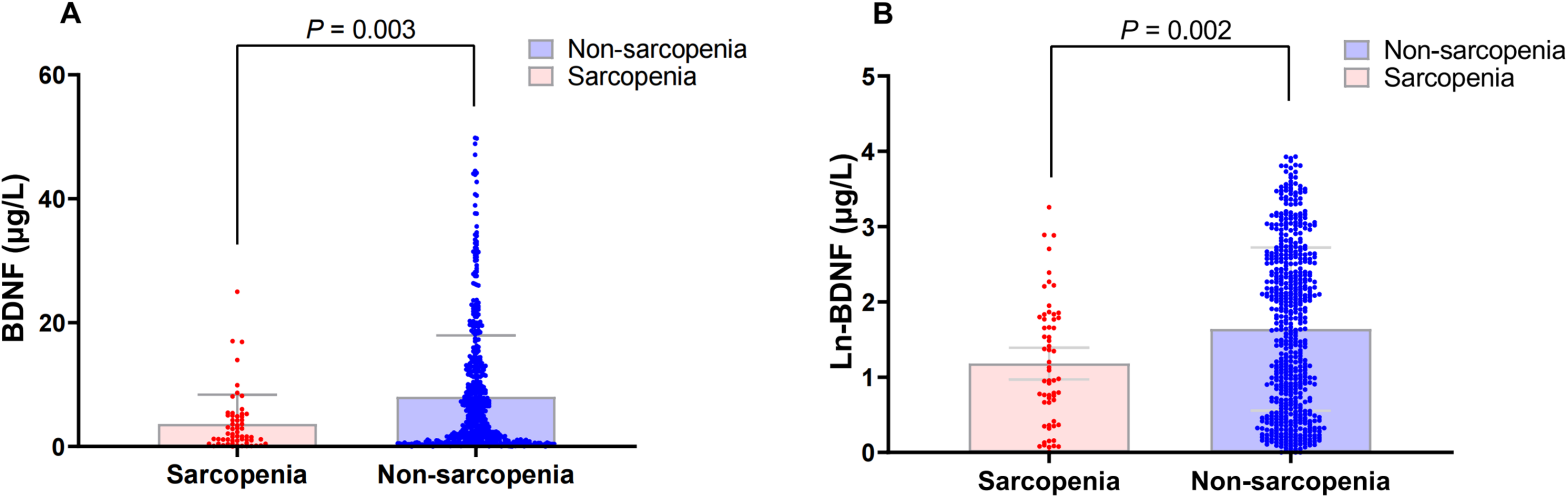
Comparison of BDNF levels between older adults with and without sarcopenia.

**FIGURE 3:**
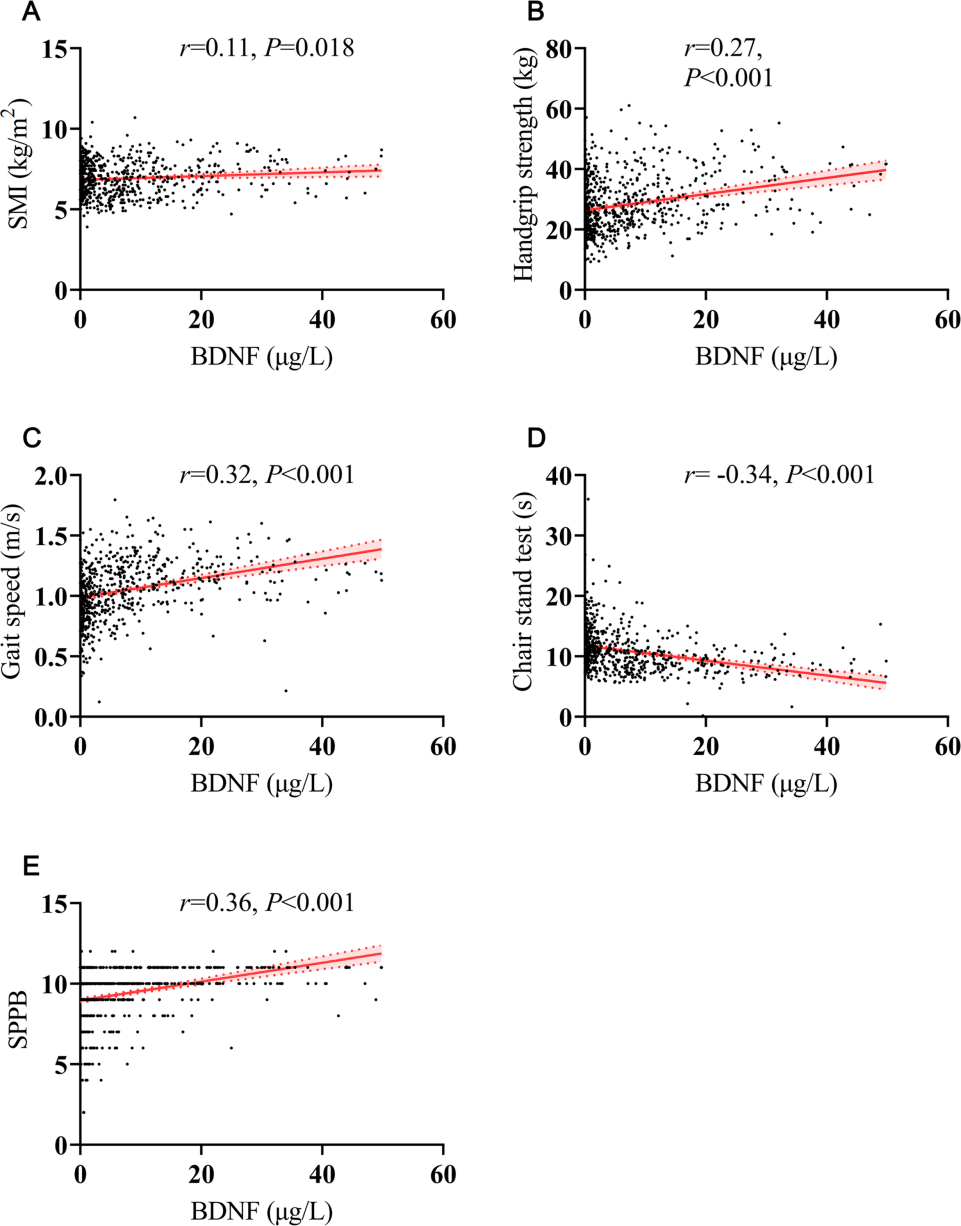
Correlations of BDNF levels with muscle mass (A), muscle strength (B) and physical function (C–E).

**FIGURE 4:**
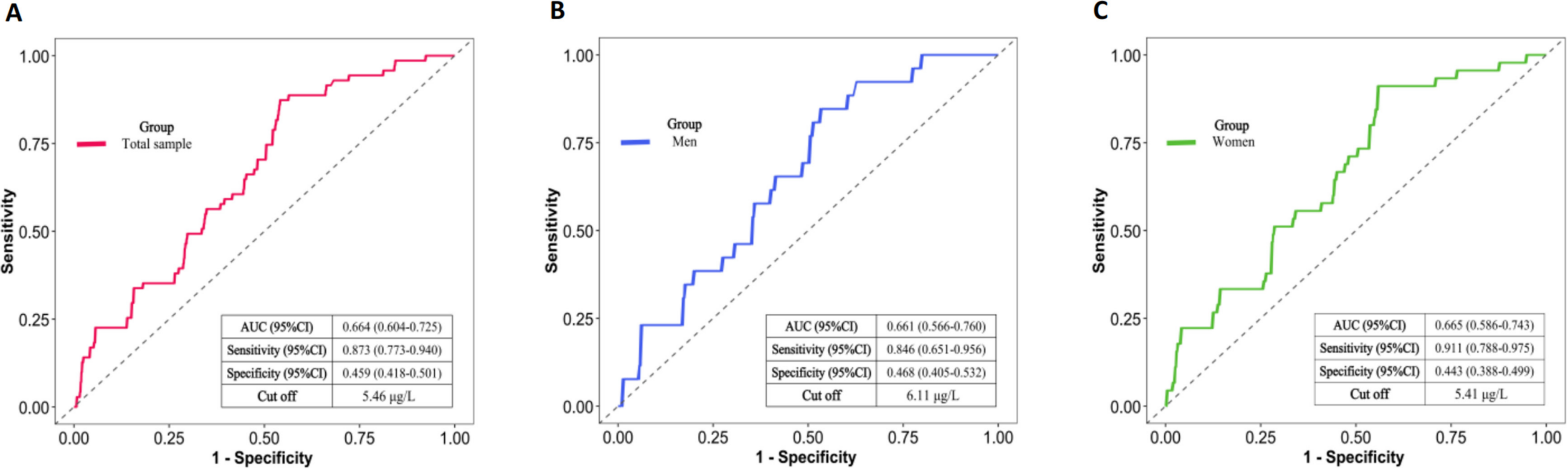
Receiver operating characteristic curves of BDNF for identifying sarcopenia in the total sample (A), men (B) and women (C).

As shown in Figure 2, older adults with sarcopenia exhibited significantly lower levels of both absolute BDNF concentration [1.82 (0.62, 4.95) vs. 4.12 (0.81, 11.46), *p* = 0.003] and ln‐transformed BDNF concentration (1.18 ± 0.81 vs. 1.64 ± 1.08, *p* = 0.002) compared with those without sarcopenia. BDNF showed mild positive correlations with SMI (*r* = 0.11, *p* = 0.018), moderate positive correlations with handgrip strength (*r* = 0.27, *p* < 0.001), gait speed (*r* = 0.32, *p* < 0.001), SPPB score (*r* = 0.36, *p* < 0.001) and a negative correlation with five‐times sit‐to‐stand time (*r* = −0.34, *p* < 0.001) (Figure 3). After adjusting for age, sex, ethnicity, marital status, smoking, drinking, habitual exercise, BMI, ADL, cognitive function, depressive symptoms and comorbidity index, ln‐transformed BDNF (as a continuous variable) was associated with a lower prevalence of sarcopenia (OR = 0.71, 95% CI: 0.63–0.81, *p* = 0.012), with the statistical power calculated at 0.87. Compared with participants in the lowest tertile of BDNF, those in the medium tertile showed no significant association with sarcopenia (OR = 0.85, 95% CI: 0.45–1.59, *p* = 0.615), whereas those in the highest tertile had 61% lower odds of sarcopenia (OR = 0.39, 95% CI: 0.16–0.91, *p* = 0.033). The statistical power for the tertile‐categorized BDNF variable in the fully adjusted model was 0.83. A significant inverse trend was observed between BDNF levels and sarcopenia in Model 3 (*P*‐trend = 0.024). These associations remained consistent across all three adjusted models, as detailed in Table 2. ROC analysis further revealed that circulating BDNF had diagnostic value for sarcopenia in the total population, with an AUC of 0.664 (95% CI: 0.604–0.725) (Figure 4). The optimal cut‐off value of BDNF for identifying sarcopenia, determined by Youden's index, was 5.46 μg/L, with a sensitivity of 0.873 (95% CI: 0.773–0.940) and a specificity of 0.459 (95% CI: 0.418–0.501). In sex‐stratified analyses, the discriminatory performance of BDNF was comparable between men and women, with an AUC of 0.661 (95% CI: 0.566–0.760) in men and 0.665 (95% CI: 0.586–0.743) in women.

**TABLE 2:** Multivariable logistic regression analysis of the association between BDNF and sarcopenia.

Groups	Model 1		Model 2		Model 3	
	OR (95% CI)	*p*	OR (95% CI)	*p*	OR (95% CI)	*p*
**As continuous variable**						
ln‐BDNF	0.54(0.31–0.84)	0.005	0.55(0.32–0.88)	0.009	0.71(0.63–0.81)	0.012
**As categorical variable**						
Q1 (< 1.46 μg/L)	Ref.		Ref.		Ref.	
Q2 (≥ 1.46 and < 7.79 μg/L)	0.91(0.49–1.64)	0.738	0.87(0.47–1.59)	0.106	0.85(0.45–1.59)	0.615
Q3 (≥ 7.79 μg/L)	0.42(0.21–0.85)	0.022	0.38(0.16–0.89)	0.026	0.39(0.16–0.91)	0.033
*P*‐trend		0.019		0.017		0.024

*Note:* Model 1: adjusting for age and sex; Model 2: additionally adjusting for ethnicity, marital status, smoking, drinking, habitual exercise and BMI; Model 3: additionally adjusting for ADL, MMSE, depressive symptom and comorbidity index.

### 3.3 Association Between BDNF and Gait Characteristics

**FIGURE 5:**
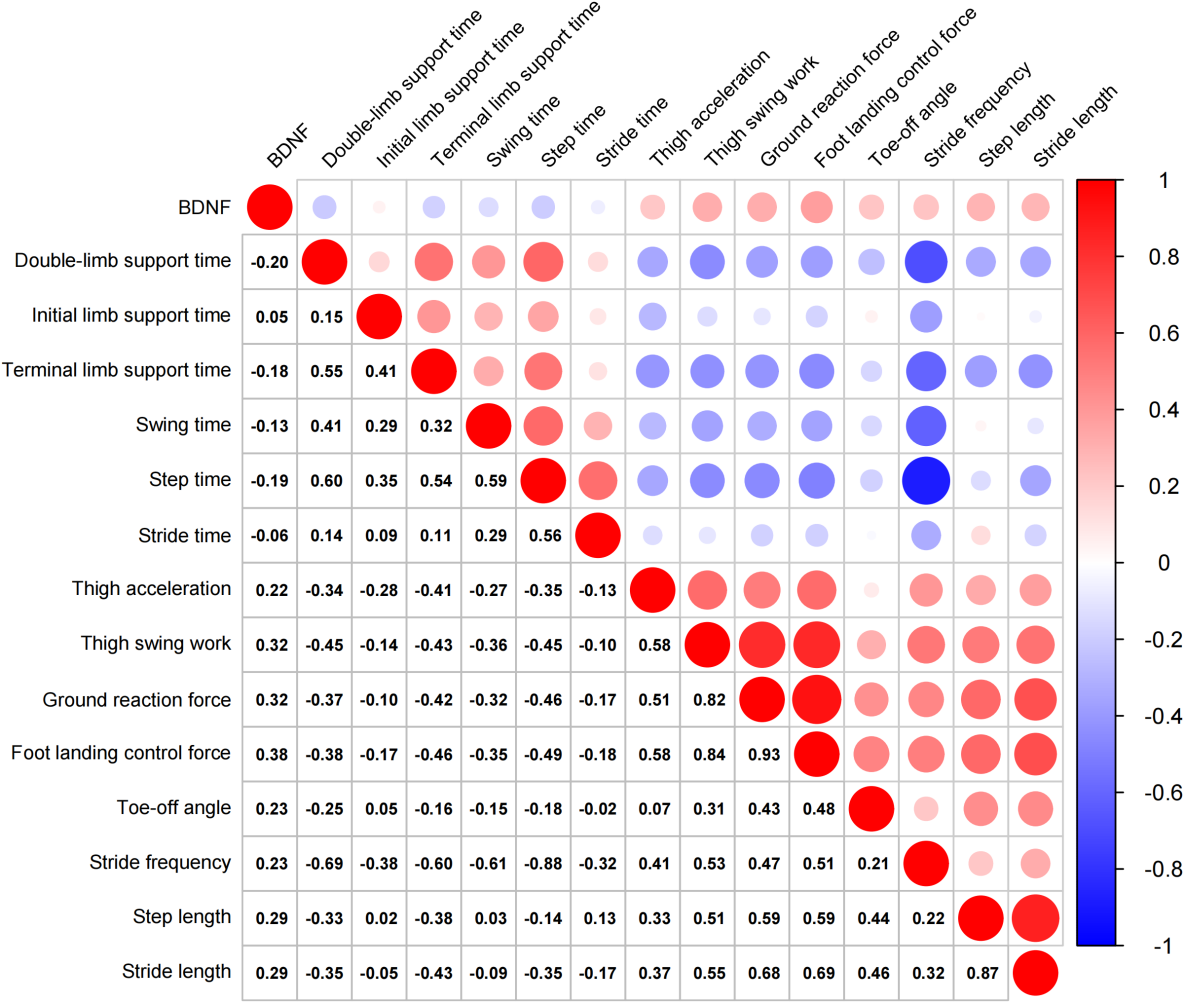
Spearman correlation coefficients between BDNF and gait parameters.

As shown in Figure 5, BDNF was negatively correlated with double‐limb support time, terminal double‐limb support time and swing time (all *p* < 0.01), with Spearman's *r* values ranging from −0.20 to −0.18. Conversely, BDNF showed positive correlations with thigh acceleration, thigh swing work, ground reaction force, foot landing control force, toe‐off angle, stride frequency, step length and stride length (all *p* < 0.001), with Spearman's *r* values ranging from 0.23 to 0.38. After adjusting for all covariates, ln‐transformed BDNF remained negatively associated with swing time (*β* = −7.833, *p* = 0.011), step time (*β* = −12.769, *p* = 0.016) and stride time (*β* = −0.026, *p* = 0.012) and positively associated with thigh acceleration (*β* = 0.066, *p* < 0.001), thigh swing work (*β* = 0.039, *p* < 0.001), ground reaction force (*β* = 0.083, *p* < 0.001), foot landing control (*β* = 0.224, *p* < 0.001), toe‐off angle (*β* = 2.061, *p* < 0.001), step frequency (*β* = 1.615, *p* < 0.001) and stride length (*β* = 0.013, *p* < 0.001). The statistical power for ln‐transformed BDNF in these fully adjusted gait parameter models ranged from 0.91 to 0.99. Results of the multivariable linear regression analysis examining the association between BDNF and gait parameters are shown in Table 3.

**TABLE 3:** Multivariable linear regression analysis of the association between BDNF and gait parameters.

Dependent variables	Model 1			Model 2			Model 3		
	*β*	SE	*p*	*β*	SE	*p*	*β*	SE	*p*
**Periodic parameters**									
Double‐limb support time	−6.409	2.459	0.019	−2.404	2.749	0.383	−1.211	2.934	0.682
Initial limb support time	0.201	0.667	0.879	0.383	0.761	0.413	0.449	0.821	0.584
Terminal limb support time	−4.889	1.342	0.001	−3.747	1.531	0.015	−3.546	2.644	0.134
Swing time	−7.794	2.444	0.002	−8.186	2.841	0.004	−7.833	3.082	0.011
Step time	−14.129	4.303	0.001	−14.258	4.877	0.004	−12.769	5.311	0.016
Stride time	−0.029	0.008	< 0.001	−0.027	0.009	0.003	−0.026	0.011	0.012
**Kinetic parameters**									
Thigh acceleration	0.079	0.016	< 0.001	0.071	0.019	< 0.001	0.066	0.021	0.001
Thigh swing work	0.048	0.008	< 0.001	0.045	0.009	< 0.001	0.039	0.009	< 0.001
Ground reaction force	0.096	0.015	< 0.001	0.092	0.016	< 0.001	0.083	0.017	< 0.001
Foot landing control force	0.283	0.035	< 0.001	0.251	0.041	< 0.001	0.224	0.043	< 0.001
Toe‐off angle	2.512	0.445	< 0.001	2.503	0.509	< 0.001	2.061	0.748	0.002
**Spatiotemporal parameters**									
Stride frequency	2.463	0.545	< 0.001	1.922	0.614	0.002	1.615	0.665	0.017
Step length	0.012	0.008	0.002	0.007	0.003	0.014	0.004	0.003	0.136
Stride length	0.025	0.006	< 0.001	0.016	0.006	0.012	0.013	0.005	0.041

*Note:* BDNF is included as a continuous variable after ln‐transformation. Model 1: adjusting for age and sex; Model 2: additionally adjusting for ethnicity, marital status, smoking, drinking and BMI; Model 3: additionally adjusting for ADL, MMSE, depressive symptom and comorbidity index.

### 3.4 Potential Mechanisms

To explore potential mechanisms through which BDNF may influence sarcopenia and gait performance, we further examined a series of inflammatory and oxidative stress markers. As shown in Table 1, older adults with sarcopenia exhibited significantly higher levels of IL‐6 [23.09 (9.65, 64.28) vs. 12.03 (1.56, 47.65), *p* = 0.004] and IL‐1*β* [23.88 (6.71, 31.02) vs. 12.66 (3.27, 20.48), *p* = 0.023] compared with those without sarcopenia. As shown in Figure S1, BDNF was significantly negatively correlated with IL‐6 (*r* = −0.42, *p* < 0.001), IL‐1*β* (*r* = −0.18, *p* < 0.001), MDA (*r* = −0.16, *p* < 0.001) and GR (*r* = −0.09, *p* = 0.032), while showing positive correlations with SOD (*r* = 0.30, *p* < 0.001) and GSH‐Px (*r* = 0.11, *p* = 0.007). Hs‐CRP and IL‐6 were significantly positively correlated with periodic parameters but negatively correlated with both kinetic and spatiotemporal parameters. In contrast, SOD and GR showed significant positive correlations with kinetic and spatiotemporal parameters. As presented in Figure S2, PLS‐SEM analysis confirmed a significant mediating role of IL‐1*β* in the association between BDNF and sarcopenia with a PM of 14.65% (95% CI: 1.65%–45.95%, *p* = 0.028) after full adjustment. No significant mediating effects were observed for other inflammatory and oxidative stress markers.

### 3.5 Sensitivity Analyses

After excluding 41 participants with ADL impairment and nine participants with MMSE score < 21, the ORs for sarcopenia of ln‐BDNF were 0.63 (95% CI: 0.46–0.86) and 0.65 (95% CI: 0.51–0.84), respectively (Tables S1–S2). Moreover, BDNF remained positively correlated with kinetic parameters and spatiotemporal parameters (Tables S3–S4). Additionally, after excluding older adults with stroke and Parkinson's disease, the associations between BDNF and sarcopenia as well as gait parameters were consistent with the main findings (Tables S5–S6). We further adjusted for medication use and found that the associations of BDNF with sarcopenia and gait parameters remained unchanged in the fully adjusted model (Tables S7–S8). Additionally, gait parameters were also *Z*‐transformed to compare the effect sizes across different indicators. The association of BDNF with terminal limb support time, swing time, step time, stride time, thigh acceleration, thigh swing work, ground reaction force, foot landing control force, toe‐off angle, stride frequency and stride length was significant (Table S9). The effect size of BDNF on kinetic parameters was greater than that on periodic and spatiotemporal parameters.

## 4 Discussion

This study revealed that higher plasma BDNF levels were independently associated with a lower prevalence of sarcopenia and superior gait performance in older adults. These findings extend the current understanding of the brain–muscle axis by linking BDNF not only to the classical components of sarcopenia but also to refined sarcopenia‐related gait parameters derived from wearable sensors.

We observed a significant inverse association between plasma BDNF levels and sarcopenia, which remained robust after adjusting for a wide range of potential confounders. Compared with the lowest tertile, participants in the highest BDNF tertile had a 61% lower odds of sarcopenia. The nonsignificant association for the medium BDNF tertile may be explained by a biological threshold effect of BDNF against sarcopenia, as well as the limited number of sarcopenia cases in our study, which reduced statistical power to detect a moderate effect. Future studies with larger sample sizes are needed to validate the dose–response relationship between circulating BDNF and sarcopenia. ROC analysis further confirmed the acceptable discriminatory value of BDNF for identifying sarcopenia. Notably, we found that circulating BDNF levels were significantly lower in the sarcopenia group than in the healthy control group, despite a wide concentration range and a certain degree of concentration overlap between the two groups. Despite our strict standardized protocols for platelet‐poor plasma preparation to minimize preanalytical bias caused by in vitro platelet activation and BDNF release, the inherent biological characteristic that the vast majority of circulating BDNF is stored in platelets in vivo cannot be eliminated. Accordingly, the wide interindividual range of BDNF levels remains a well‐documented and consistent finding in large community‐based population studies of older adults. This overlap can be attributed to the fact that circulating BDNF is modulated by various factors beyond skeletal muscle‐related indicators. Although BDNF has been identified as a biomarker associated with sarcopenia in the current study, its diagnostic specificity remains to be verified in future studies. Specifically, our ROC analysis observed a moderate AUC for BDNF in sarcopenia detection, with significantly higher sensitivity than specificity, which also explains the need for further validation of its discriminatory performance.

BDNF demonstrated moderate positive correlation with handgrip strength, gait speed and SPPB score, whereas its correlations with appendicular skeletal muscle index were weak. This pattern suggests that the protective role of BDNF against sarcopenia may operate primarily through preserving neuromuscular function and quality of muscle contraction rather than by merely increasing muscle mass. We further compared the absolute and ln‐transformed BDNF concentrations across nonsarcopenic, possible sarcopenia and confirmed sarcopenia groups. Multiple comparisons revealed that BDNF levels were significantly lower in participants with possible or confirmed sarcopenia than in healthy nonsarcopenic controls, whereas no significant difference was observed between the possible sarcopenia and sarcopenia subgroups (Figure S3). This finding may indicate the temporal and mechanistic characteristics of BDNF in sarcopenia. Possible sarcopenia is characterized by low muscle strength and poor physical function, marking the early prodromal stage of sarcopenia. The significant reduction in BDNF already observed at this stage demonstrates that BDNF alterations are an early event in sarcopenia pathogenesis, rather than a secondary consequence of muscle mass loss. Additionally, the relatively weak correlation between BDNF and muscle mass further supports that BDNF exerts its primary biological role by preserving neuromuscular function and muscle contraction quality, rather than by regulating muscle mass accrual. Furthermore, multiparametric gait analysis precisely captured locomotor impairments associated with sarcopenia. As shown in Table 1, individuals with sarcopenia exhibited significant reductions in key kinetic and spatiotemporal parameters. Importantly, higher BDNF levels were correlated with better performance across these diverse gait metrics. Collectively, these results provide convergent evidence that BDNF supports more efficient neural control of skeletal muscle movement.

Our findings are consistent with several clinical studies that underscore BDNF's association with muscle function and muscle mass. Satoru et al. reported significantly lower BDNF levels in Japanese haemodialysis patients with severe sarcopenia and frailty, where BDNF correlated positively with muscle strength and physical performance, but not with body mass index or appendicular skeletal muscle mass [13]. Similarly, Daniel et al. found that among patients with disease‐related malnutrition, those with higher BDNF levels had an 84% reduced risk of sarcopenia after adjusting for age, sex, BMI and energy intake [14]. Another Japanese study of 60 heart failure patients demonstrated that serum BDNF levels were associated with skeletal muscle function independent of muscle mass [28]. However, the relationship appears complex, as evidenced by a recent study of 246 adults aged 50–82 years, which reported a positive association between elevated plasma BDNF concentrations and sarcopenia [16]. The authors postulated that higher BDNF might reflect a compensatory upregulation in response to ongoing neuromuscular decline. Collectively, these discrepant findings suggest a dual potential role for BDNF in sarcopenia. Elevated BDNF levels may represent an adaptive, compensatory response to facilitate neuromuscular remodelling, whereas BDNF deficiency could directly contribute to the advancement of muscle atrophy and neurodegeneration. BDNF is increasingly recognized as a pivotal molecule bridging the neural and muscular systems, yet its precise mechanisms and potential as an interventional target warrant further elucidation.

This study provides novel evidence for significant associations between BDNF and multidimensional gait parameters. A key methodological advancement lies in the use of wearable sensors, moving beyond conventional assessments like gait speed to capture comprehensive biomechanical profiles. Although Spearman's correlation analyses revealed weak‐to‐moderate associations between BDNF levels and gait parameters, BDNF's correlation with these metrics supports its value as an auxiliary biomarker for the early screening of sarcopenia‐related gait decline. It should also be noted that BDNF is most likely a marker of upstream systemic physiological processes rather than a direct mediator of muscle health. After adjusting for multiple health status confounders, BDNF levels showed negative correlations with swing time, step time and stride time, while demonstrating positive correlations with thigh acceleration, thigh swing work, ground reaction force, foot landing control, toe‐off angle, step frequency and stride length (all *p* < 0.05). After standardizing gait parameters, we further found that the associations of BDNF with kinetic parameters were stronger than those with spatiotemporal and periodic parameters. Previous research on the relationship between BDNF and gait characteristics has primarily focused on neurodegenerative populations, yielding conflicting results. A Taiwanese study reported that lower plasma BDNF levels significantly correlated with severity of postural instability and gait difficulty in Parkinson's disease patients [29]. Another recent intervention study found that multimodal exercise was associated with improved cognitive and motor function in Alzheimer's disease patients, accompanied by increased plasma BDNF and decreased interleukin‐2 levels [30]. Conversely, Nathaniel et al. found no significant association between BDNF Val66Met polymorphism and gait dysfunction in 30 Parkinson's patients [31]. In a study of 20 people with multiple sclerosis, Felipe et al. observed moderate correlations between BDNF and gait variables; however, these associations did not remain statistically significant after Bonferroni correction [26]. These nonsignificant findings may stem from methodological limitations, such as small sample sizes, limited statistical power and insufficiently sensitive gait evaluation. In contrast, we observed a significant positive association between BDNF and gait performance in a substantial community‐based sample, which remained robust after adjusting for multiple covariates.

The mechanisms through which BDNF influences sarcopenia and gait characteristics are complex. BDNF appears to directly drive muscular adaptation and plays an important role in improving muscle plasticity and function. Lower BDNF levels are closely associated with reduced protein synthesis as well as increased degradation [32]. Furthermore, BDNF is involved in regulating mitochondrial function, which is essential for energy supply, contraction efficiency and protecting muscle cells from damage [33]. At the neuromuscular level, BDNF is indispensable for the integrity and function of the NMJ, whose degeneration is a key factor in the development of sarcopenia and gait impairment [34]. More specifically, through its TrkB receptor signalling pathway, BDNF likely contributes to the survival of motor neurons and the regeneration of axons, thereby influencing muscle innervation and overall function [35]. BDNF also exerts protective effects on cardiac muscle via multiple targets, including vascular smooth muscle cells and endothelial cells, thereby benefiting both systolic and diastolic heart function [20, 36]. This suggests a potential link between BDNF's role in cardiovascular health and physical mobility, together contributing to the maintenance of overall motor homeostasis.

As summarized in Figures S1–S2, our findings provide preliminary evidence that the association between circulating BDNF levels and muscle wasting and gait dysfunction may be linked to inflammatory and oxidative stress pathways. Specifically, markers such as IL‐6, IL‐1*β*, SOD and GR may partially explain the physiological association of BDNF with skeletal muscle health. Mediation analyses revealed that IL‐1*β* explained 14.65% of the total association between BDNF and sarcopenia, suggesting that downregulation of proinflammatory IL‐1*β* signalling may be a key pathway linking BDNF to skeletal muscle health. No statistically significant mediating effects of SOD and GSH‐Px were detected, likely due to limited sample size; however, the negative mediated effect proportion for these markers indicated a potential suppression effect, which needs further validation in future studies. Chronic inflammation can reduce BDNF levels, whereas BDNF itself may inhibit neuroinflammation by modulating microglial activation [37]. In Alzheimer's disease models, hippocampal BDNF expression negatively correlates with neuroinflammatory markers and pathological proteins [38]. In Parkinson's disease patients, inflammation and oxidative stress influence BDNF levels by altering neuroplasticity, which is associated with the severity of motor symptoms [39]. Basic research further supports these connections. Deficiency of muscle‐derived BDNF leads to inflammatory myopathy through enhanced oxidative stress, necroptosis and pyroptosis. A recent study by Pang and colleagues demonstrated that muscle‐specific BDNF knockout mice exhibited extensive myocyte necrosis, mononuclear cell infiltration and mitochondrial dysfunction, changes that are directly linked to elevated oxidative stress [10]. Moreover, oxidative stress acts as an early trigger of muscle atrophy by impairing mitochondrial function and disrupting the balance between protein synthesis and degradation. BDNF has also been shown to counteract this process by activating the AMPK*α*–PGC1*α* pathway, improving mitochondrial function and thereby ameliorating motor deficits [20]. Together, these foundational studies and our exploratory results highlight the central role of the interplay between BDNF, oxidative stress and inflammation in the pathophysiology of muscle deterioration.

This study has several limitations. First, the cross‐sectional design prevents any determination of causality. Longitudinal studies are needed to establish whether BDNF levels can predict future changes in sarcopenia status and gait performance. Second, the generalizability of our findings may be limited, as all participants were recruited from urban areas in China. Future studies enrolling more diverse populations and applying different sarcopenia diagnostic criteria are therefore warranted. Additionally, participants who are able to complete wearable device‐based gait measurements are generally healthier physically and neurologically than the general older population, which may introduce selection bias. However, the prevalence of sarcopenia in our sample was 10.06%, which is close to the reported prevalence among community‐dwelling older adults in China. Third, although we examined several inflammatory and oxidative stress biomarkers theoretically associated with BDNF and provided population‐level mechanistic insights, the set of biomarkers included was not exhaustive. Further research incorporating multiomics approaches and experimental validation is necessary to more comprehensively elucidate the underlying mechanisms. Fourth, the measured plasma BDNF cannot be attributed to a specific tissue source, such as the brain or skeletal muscle. The relatively small sample size of the sarcopenia group may also affect the distribution characteristics of BDNF levels and the robustness of our findings. Elucidating the precise role of BDNF from different cellular origins and its subtypes in sarcopenia requires further investigation in large‐sample prospective cohorts and animal models.

## 5 Conclusion

This study demonstrates that higher plasma BDNF levels are associated with a lower prevalence of sarcopenia in community‐dwelling older adults. Using wearable sensors, we established correlations between BDNF and superior gait performance, encompassing both kinetic and spatiotemporal parameters. Our findings reveal concurrent correlations between BDNF and inflammatory biomarkers, supporting its potential as a biomarker of neuromuscular function. These results advance the understanding of the brain–muscle axis in skeletal muscle health and provide a reference for the early identification of sarcopenia.

## Funding

This study is supported by the National High Level Hospital Clinical Research Funding (grant numbers: BJ‐2025‐253, BJ‐2019‐200, BJ‐2024‐157, BJ‐2025‐240 and BJ‐2022‐133).

## Ethics Statement

The study protocol was approved by the Ethics Committee of Beijing Hospital (2022BJYYEC‐263‐02), and written informed consent was obtained from all participants.

## Consent

The authors have nothing to report.

## Conflicts of Interest

The authors declare no conflicts of interest.

## Supporting information


**Figure S1:** Correlations of inflammatory and oxidative stress markers with sarcopenia status and gait parameters.
**Figure S2:** Mediation analyses of the association between BDNF and sarcopenia via inflammatory and oxidative stress markers.
**Figure S3:** Comparison of BDNF concentrations across nonsarcopenic, possible sarcopenia and confirmed sarcopenia groups.
**Table S1:** Multivariable logistic regression analysis of the association between BDNF and sarcopenia after excluding 41 participants with ADL impairment.
**Table S2:** Multivariable logistic regression analysis of the association between BDNF and sarcopenia after excluding nine participants with MMSE score < 21.
**Table S3:** Multivariable linear regression analysis of the association between BDNF and gait parameters after excluding 41 participants with ADL impairment.
**Table S4:** Multivariable linear regression analysis of the association between BDNF and gait parameters after excluding nine participants with MMSE score < 21.
**Table S5:** Multivariable logistic regression analysis of the association between BDNF and sarcopenia after excluding 28 participants with stroke and 1 participant Parkinson's disease.
**Table S6:** Multivariable linear regression analysis of the association between BDNF and gait parameters after excluding 28 participants with stroke and one participant Parkinson's disease.
**Table S7:** Multivariable logistic regression analysis of the association between BDNF and sarcopenia after additionally adjusting for polypharmacy.
**Table S8:** Multivariable linear regression analysis of the association between BDNF and gait parameters after additionally adjusting for polypharmacy.
**Table S9:** Multivariable linear regression analysis of the association between BDNF and *Z*‐scores of gait parameters.

## Data Availability

The datasets analysed during the current study are available from the corresponding author on reasonable request.
