# Supplementary material for: Brain‐Derived Neurotrophic Factor, Sarcopenia and Digital Gait Characteristics in Older Adults: Insights Into the Brain–Muscle Axis
Source: J Cachexia Sarcopenia Muscle. 2026 Jun 30;17(4):e70325. doi: 10.1002/jcsm.70325 (PMC13315818; doi:10.1002/jcsm.70325)
Supplement: Supplementary file 1 — Figure S1: Correlations of inflammatory and oxidative stress markers with sarcopenia status and gait parameters. Figure S2: Mediation analyses of the association between BDNF and sarcopenia via inflammatory and oxidative stress markers. Figure S3: Comparison of BDNF concentrations across nonsarcopenic, possible sarcopenia and confirmed sarcopenia groups. Table S1: Multivariable logistic regression analysis of the association between BDNF and sarcopenia after excluding 41 participants with ADL impairment. Table S2: Multivariable logistic regression analysis of the association between BDNF and sarcopenia after excluding nine participants with MMSE score < 21. Table S3: Multivariable linear regression analysis of the association between BDNF and gait parameters after excluding 41 participants with ADL impairment. Table S4: Multivariable linear regression analysis of the association between BDNF and gait parameters after excluding nine participants with MMSE score < 21. Table S5: Multivariable logistic regression analysis of the association between BDNF and sarcopenia after excluding 28 participants with stroke and 1 participant Parkinson's disease. Table S6: Multivariable linear regression analysis of the association between BDNF and gait parameters after excluding 28 participants with stroke and one participant Parkinson's disease. Table S7: Multivariable logistic regression analysis of the association between BDNF and sarcopenia after additionally adjusting for polypharmacy. Table S8: Multivariable linear regression analysis of the association between BDNF and gait parameters after additionally adjusting for polypharmacy. Table S9: Multivariable linear regression analysis of the association between BDNF and Z‐scores of gait parameters. [file JCSM-17-e70325-s001.docx]

**Supplementary materials**


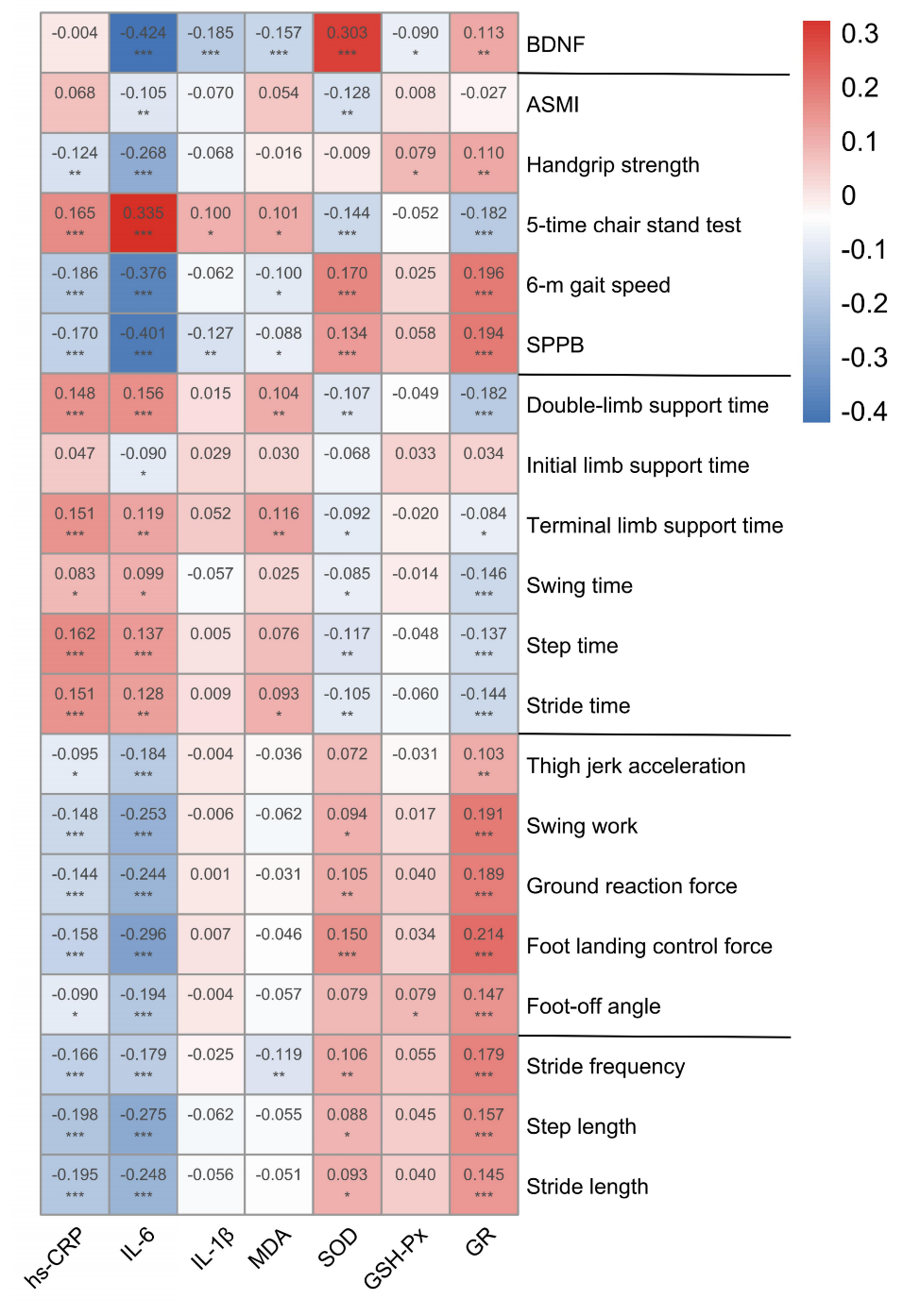


**Supplementary Figure 1. Correlations of inflammatory and oxidative stress markers with sarcopenia status and gait parameters**


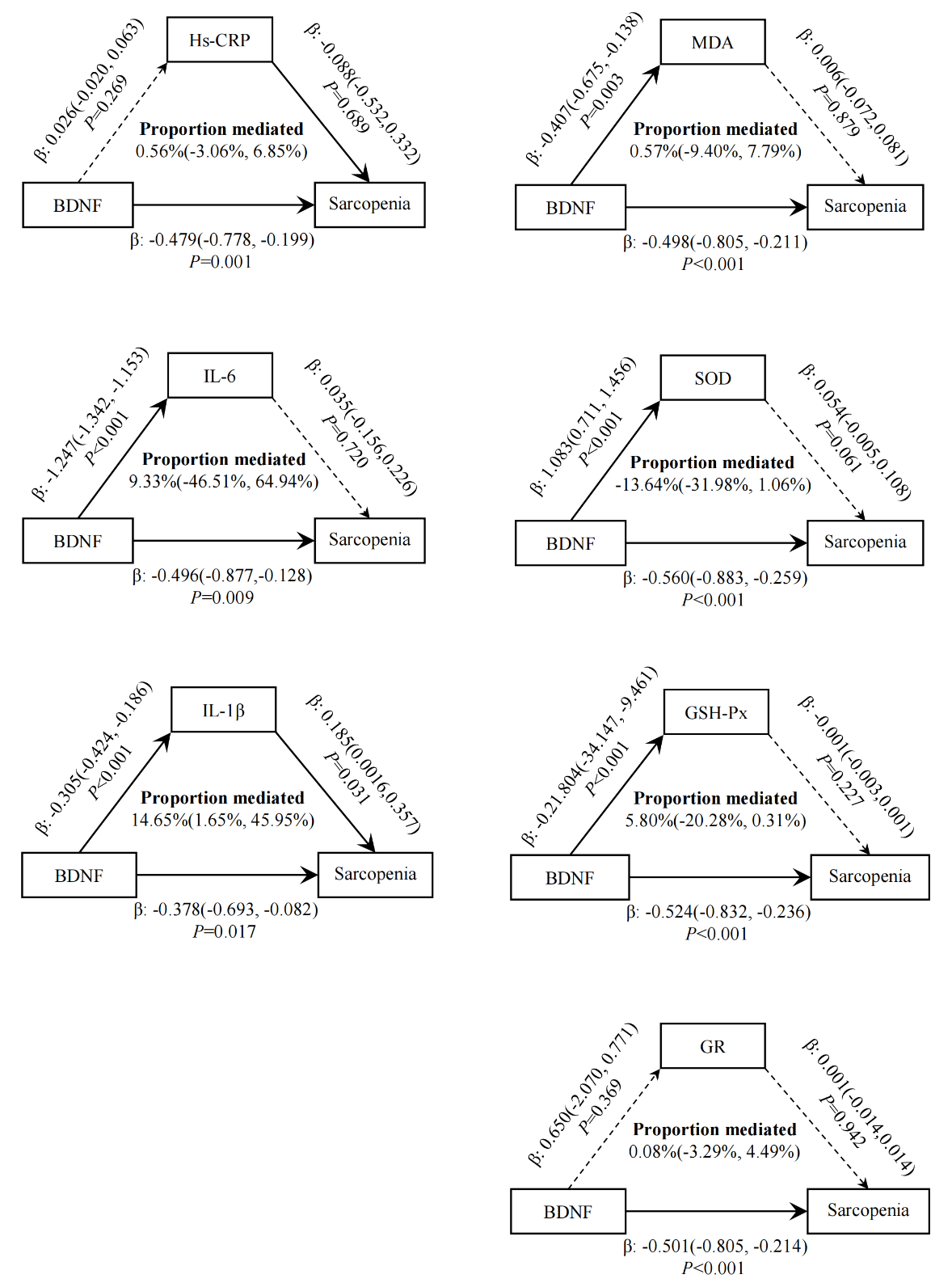


**Supplementary Figure 2. Mediation analyses of the association between BDNF and sarcopenia via inflammatory and oxidative stress markers**


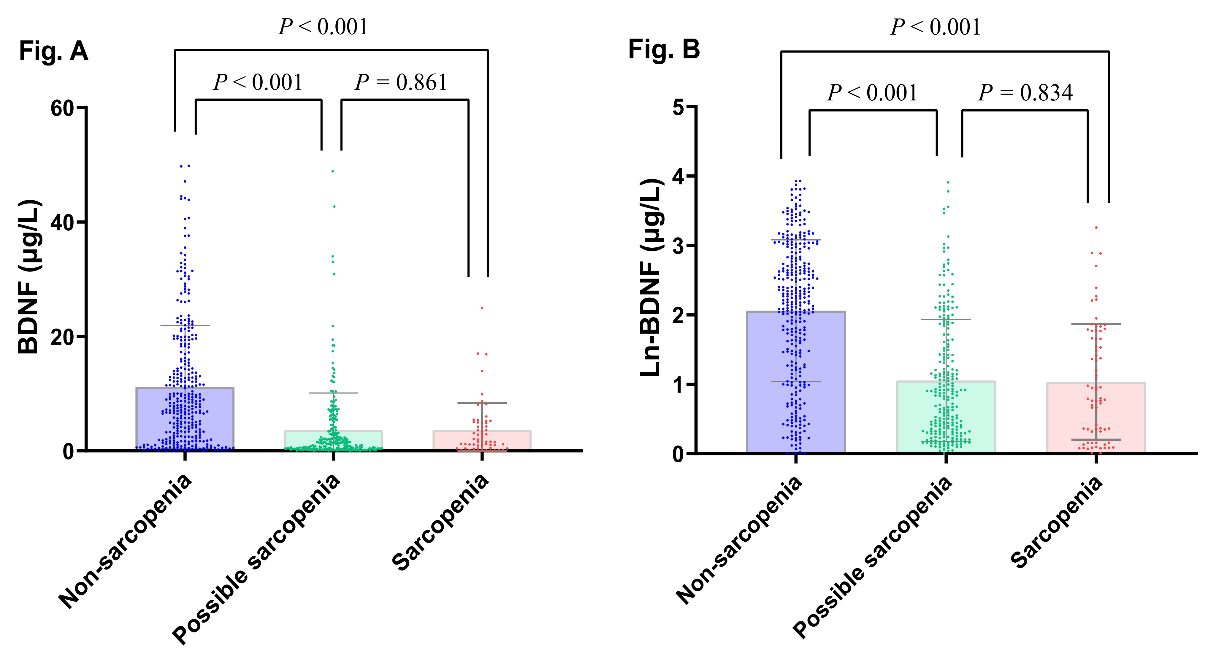


**Supplementary Figure 3. Comparison of BDNF concentrations across non-sarcopenic, possible sarcopenia, and confirmed sarcopenia groups**

**Supplementary Table 1.** Multivariable logistic regression analysis of the association between BDNF and sarcopenia after excluding 41 participants with ADL impairment

| **Groups** | **Fully adjusted model** | |
| --- | --- | --- |
|  | OR (95% CI) | *P*-value |
| **As continuous variable** |  |  |
| ln-BDNF | 0.63(0.46-0.87) | 0.005 |
| **As categorical variable** |  |  |
| Q1 (< 1.39 μg/L) | Ref. |  |
| Q2 (≥ 1.39 and < 7.91 μg/L) | 0.84(0.43-1.65) | 0.621 |
| Q3 (≥ 7.91 μg/L) | 0.32(0.13-0.77) | 0.012 |
| *P*-trend |  | 0.010 |

Adjusting for age, sex, ethnicity, marital status, smoking, drinking, habitual exercise, BMI, MMSE, depressive symptom, and comorbidity index.

**Supplementary Table 2.** Multivariable logistic regression analysis of the association between BDNF and sarcopenia after excluding 9 participants with MMSE score < 21

| **Groups** | **Fully adjusted model** | |
| --- | --- | --- |
|  | OR (95% CI) | *P*-value |
| **As continuous variable** |  |  |
| ln-BDNF | 0.65(0.51-0.84) | 0.003 |
| **As categorical variable** |  |  |
| Q1 (< 1.41 μg/L) | Ref. |  |
| Q2 (≥ 1.41 and < 7.78 μg/L) | 0.92(0.48-1.75) | 0.795 |
| Q3 (≥ 7.78 μg/L) | 0.31(0.19-0.46) | 0.008 |
| *P*-trend |  | 0.009 |

Adjusting for age, sex, ethnicity, marital status, smoking, drinking, habitual exercise, BMI, ADL, MMSE, depressive symptom, and comorbidity index.

**Supplementary Table 3.** Multivariable linear regression analysis of the association between BDNF and gait parameters after excluding 41 participants with ADL impairment

| **Dependent variables** | **Fully adjusted model** | | |
| --- | --- | --- | --- |
|  | β | SE | *P*-value |
| **Periodic parameters** |  |  |  |
| Double-limb support time | 0.223 | 2.698 | 0.934 |
| Initial limb support time | 0.092 | 0.761 | 0.904 |
| Terminal limb support time | -3.229 | 1.525 | 0.035 |
| Swing time | -6.586 | 2.753 | 0.017 |
| Step time | -9.535 | 4.852 | 0.051 |
| Stride time | -0.019 | 0.009 | 0.036 |
| **Kinetic parameters** |  |  |  |
| Thigh acceleration | 0.062 | 0.019 | 0.001 |
| Thigh swing work | 0.032 | 0.009 | 0.001 |
| Ground reaction force | 0.072 | 0.016 | <0.001 |
| Foot landing control force | 0.202 | 0.039 | 0.001 |
| Toe-off angle | 2.224 | 0.509 | <0.001 |
| **Spatiotemporal parameters** |  |  |  |
| Stride frequency | 1.296 | 0.609 | 0.034 |
| Step length | 0.005 | 0.003 | 0.094 |
| Stride length | 0.011 | 0.006 | 0.061 |

Adjusting for age, sex, ethnicity, marital status, smoking, drinking, habitual exercise, ADL, BMI, MMSE, depressive symptom, and comorbidity index.

**Supplementary Table 4.** Multivariable linear regression analysis of the association between BDNF and gait parameters after excluding 9 participants with MMSE score < 21

| **Dependent variables** | **Fully adjusted model** | | |
| --- | --- | --- | --- |
|  | β | SE | *P*-value |
| **Periodic parameters** |  |  |  |
| Double-limb support time | -1.118 | 2.712 | 0.869 |
| Initial limb support time | -0.229 | 0.757 | 0.761 |
| Terminal limb support time | -3.941 | 1.535 | 0.011 |
| Swing time | -7.613 | 2.781 | 0.006 |
| Step time | -11.17 | 4.916 | 0.023 |
| Stride time | -0.023 | 0.009 | 0.017 |
| **Kinetic parameters** |  |  |  |
| Thigh acceleration | 0.064 | 0.019 | <0.001 |
| Thigh swing work | 0.036 | 0.009 | <0.001 |
| Ground reaction force | 0.076 | 0.017 | <0.001 |
| Foot landing control force | 0.211 | 0.061 | 0.001 |
| Toe-off angle | 2.157 | 0.515 | 0.001 |
| **Spatiotemporal parameters** |  |  |  |
| Stride frequency | 1.462 | 0.616 | 0.018 |
| Step length | 0.005 | 0.003 | 0.075 |
| Stride length | 0.012 | 0.006 | 0.036 |

Adjusting for age, sex, ethnicity, marital status, smoking, drinking, habitual exercise, ADL, BMI, MMSE, depressive symptom, and comorbidity index.

**Supplementary Table 5.** Multivariable logistic regression analysis of the association between BDNF and sarcopenia after excluding 28 participants with stroke and 1 participant Parkinson’s disease

| **Groups** | **Fully adjusted model** | |
| --- | --- | --- |
|  | OR (95% CI) | *P*-value |
| **As continuous variable** |  |  |
| ln-BDNF | 0.66(0.47-0.89) | 0.010 |
| **As categorical variable** |  |  |
| Q1 (< 1.47 μg/L) | Ref. |  |
| Q2 (≥ 1.47 and < 7.66 μg/L) | 0.86(0.44-1.64) | 0.644 |
| Q3 (≥ 7.66 μg/L) | 0.34(0.15-0.83) | 0.018 |
| *P*-trend |  | 0.012 |

Adjusting for age, sex, ethnicity, marital status, smoking, drinking, habitual exercise, BMI, ADL, MMSE, depressive symptom, and comorbidity index.

**Supplementary Table 6.** Multivariable linear regression analysis of the association between BDNF and gait parameters after excluding 28 participants with stroke and 1 participant Parkinson’s disease

| **Dependent variables** | **Fully adjusted model** | | |
| --- | --- | --- | --- |
|  | β | SE | *P*-value |
| **Periodic parameters** |  |  |  |
| Double-limb support time | 0.759 | 2.761 | 0.787 |
| Initial limb support time | -0.076 | 0.773 | 0.921 |
| Terminal limb support time | -3.076 | 1.572 | 0.051 |
| Swing time | -7.269 | 2.871 | 0.012 |
| Step time | -10.528 | 5.049 | 0.038 |
| Stride time | -0.022 | 0.009 | 0.026 |
| **Kinetic parameters** |  |  |  |
| Thigh acceleration | 0.063 | 0.019 | 0.001 |
| Thigh swing work | 0.034 | 0.009 | <0.001 |
| Ground reaction force | 0.075 | 0.017 | <0.001 |
| Foot landing control force | 0.212 | 0.041 | <0.001 |
| Toe-off angle | 2.416 | 0.622 | 0.001 |
| **Spatiotemporal parameters** |  |  |  |
| Stride frequency | 1.318 | 0.629 | 0.037 |
| Step length | 0.005 | 0.003 | 0.062 |
| Stride length | 0.014 | 0.006 | 0.027 |

Adjusting for age, sex, ethnicity, marital status, smoking, drinking, habitual exercise, ADL, BMI, MMSE, depressive symptom, and comorbidity index.

**Supplementary Table 7.** Multivariable logistic regression analysis of the association between BDNF and sarcopenia after additionally adjusting for polypharmacy

| **Groups** | **Fully adjusted model** | |
| --- | --- | --- |
|  | OR (95% CI) | *P*-value |
| **As continuous variable** |  |  |
| ln-BDNF | 0.67(0.48-0.91) | 0.009 |
| **As categorical variable** |  |  |
| Q1 (< 1.46 μg/L) | Ref. |  |
| Q2 (≥ 1.46 and < 7.79 μg/L) | 0.89(0.48-1.74) | 0.662 |
| Q3 (≥ 7.79 μg/L) | 0.31(0.13-0.80) | 0.011 |
| *P*-trend |  | 0.015 |

Adjusting for age, sex, ethnicity, marital status, smoking, drinking, habitual exercise, BMI, ADL, MMSE, depressive symptom, comorbidity index, and polypharmacy. Polypharmacy is categorized by the number of daily medications use (0, 1, 2, and ≥3)

**Supplementary Table 8.** Multivariable linear regression analysis of the association between BDNF and gait parameters after additionally adjusting for polypharmacy

| **Dependent variables** | **Fully adjusted model** | | |
| --- | --- | --- | --- |
|  | β | SE | *P*-value |
| **Periodic parameters** |  |  |  |
| Double-limb support time | -1.191 | 2.956 | 0.687 |
| Initial limb support time | 0.588 | 0.816 | 0.471 |
| Terminal limb support time | -3.582 | 1.649 | 0.031 |
| Swing time | -8.882 | 3.066 | 0.004 |
| Step time | -14.239 | 5.283 | 0.008 |
| Stride time | -0.029 | 0.011 | 0.009 |
| **Kinetic parameters** |  |  |  |
| Thigh acceleration | 0.065 | 0.021 | 0.002 |
| Thigh swing work | 0.039 | 0.010 | <0.001 |
| Ground reaction force | 0.082 | 0.018 | <0.001 |
| Foot landing control force | 0.227 | 0.044 | <0.001 |
| Toe-off angle | 2.082 | 0.622 | 0.001 |
| **Spatiotemporal parameters** |  |  |  |
| Stride frequency | 1.782 | 0.665 | 0.010 |
| Step length | 0.005 | 0.003 | 0.114 |
| Stride length | 0.014 | 0.007 | 0.038 |

Adjusting for age, sex, ethnicity, marital status, smoking, drinking, habitual exercise, BMI, ADL, MMSE, depressive symptom, comorbidity index, and polypharmacy. Polypharmacy is categorized by the number of daily medications use (0, 1, 2, and ≥3)

**Supplementary Table 9.** Multivariable linear regression analysis of the association between BDNF and Z-scores of gait parameters

| **Dependent variables** | **Fully adjusted model** | | |
| --- | --- | --- | --- |
|  | β | SE | *P*-value |
| **Periodic parameters** |  |  |  |
| Double-limb support time (Z score) | -0.019 | 0.046 | 0.681 |
| Initial limb support time (Z score) | 0.033 | 0.049 | 0.506 |
| Terminal limb support time (Z score) | -0.104 | 0.048 | 0.031 |
| Swing time (Z score) | -0.132 | 0.049 | 0.008 |
| Step time (Z score) | -0.124 | 0.048 | 0.011 |
| Stride time (Z score) | -0.131 | 0.048 | 0.008 |
| **Kinetic parameters** |  |  |  |
| Thigh acceleration (Z score) | 0.156 | 0.050 | 0.002 |
| Thigh swing work (Z score) | 0.195 | 0.046 | <0.001 |
| Ground reaction force (Z score) | 0.219 | 0.045 | <0.001 |
| Foot landing control force (Z score) | 0.234 | 0.045 | <0.001 |
| Toe-off angle (Z score) | 0.154 | 0.046 | 0.003 |
| **Spatiotemporal parameters** |  |  |  |
| Stride frequency (Z score) | 0.123 | 0.047 | 0.009 |
| Step length (Z score) | 0.071 | 0.042 | 0.093 |
| Stride length (Z score) | 0.092 | 0.044 | 0.036 |

Adjusting for age, sex, ethnicity, marital status, smoking, drinking, habitual exercise, ADL, BMI, MMSE, depressive symptom, and comorbidity index.
